# CHANGE IN EXECUTIVE FUNCTION AFTER AN INNOVATIVE PHYSICAL THERAPY PROGRAM AMONG VETERANS WITH SLOW GAIT SPEED

**DOI:** 10.1093/geroni/igae098.1458

**Published:** 2024-12-31

**Authors:** Elisa Ogawa, Rebekah Harris, Rachel Ward, Mary Palleschi, Ildiko Halasz, William Milberg, Jonathan Bean

**Affiliations:** VA Boston Healthcare System, Boston, Massachusetts, United States; Boston VA, Boston, Massachusetts, United States; VA Boston Healthcare System, Boston, Massachusetts, United States; VA Boston Healthcare System, Jamaica Plain, Massachusetts, United States; Primary Care/VABHS, Boston, Massachusetts, United States; Harvard Medical School, Boston, Massachusetts, United States; Harvard Medical School, Boston, Massachusetts, United States

## Abstract

While gait and cognition are independent predictors of age-related adverse outcomes, evidence suggests that they are related. Thus, targeting slow gait speed may have broad effects including improvements in cognition. This study examined the effects of Live Long Walk Strong (LLWS), a novel Physical Therapy program designed to improve gait speed, on executive function among Veterans with slow gait speed. This study was a preplanned secondary analysis among the first 72 participants recruited into an ongoing Randomized Controlled Trial among middle aged to older Veterans (≥50 years) with slow walking speed. Participants were randomized to either 8-week LLWS or 8-week wait-list control. Participants’ executive function was measured using Delis-Kaplan Executive Function System Verbal Fluency subtests (Letter Fluency, Category Fluency, Category Switching). Fifty-one participants (mean age 72, 92% men, 84% White) were included in the analyses (LLWS n=27; wait-list control: n=24). We observed clinically and statistically significant changes in Category Fluency between LLWS and wait-list controls (LLWS: pre: 31.44 ± 7.92, post: 34.78 ± 8.12; wait-list control: Pre: 34.21 ± 8.54; Post: 30.83 ± 7.89, p< 0.0001), favoring the LLWS. There were no statistically or clinically significant group differences in Letter Fluency or Category Switching between LLWS and wait-list controls. LLWS may improve aspects of executive function specifically the effortful retrieval process among middle aged to older Veterans with slow gait speed.

